# Hidden leprosy in a low-endemic area in southern Brazil: changes in endemicity following an active search

**DOI:** 10.1016/j.bjid.2024.103853

**Published:** 2024-07-22

**Authors:** Bruno Vitiritti, Filipe Rocha Lima, Nara Tescke de Castilho, Lincon Bordignon Somensi, Rosana Cláudio Silva Ogoshi

**Affiliations:** a Universidade Alto Vale do Rio do Peixe, Departamento de Medicina, Caçador, SC, Brasil; b Universidade de São Paulo, Departamento de Clínica Médica, Ribeirão Preto, SP, Brasil; c Vigilância Epidemiológica de Caçador, Caçador, SC, Brasil; d Universidade Alto Vale do Rio do Peixe, Programa de Pós-Graduação em Desenvolvimento e Sociedade, Caçador, SC, Brasil

**Keywords:** Leprosy, Spatial analysis, Health profile, Health information systems

## Abstract

**Background:**

Leprosy, a neglected tropical disease, is reported in over 120 countries, with upwards of 200,000 new cases annually. This Cross-Sectional Cohort Study aimed to delineate the epidemiological profile of leprosy in a low-endemic area in southern Brazil, both before and after implementing an active search strategy.

**Methods:**

The study examined two surveillance periods in Caçador, Santa Catarina, Brazil. The active search strategy was carried out through the application of the LSQ by the community health workers as a screening and detection tool for new cases of leprosy and this was compared with passive case detection. The first spanned from 2014 to 2020, and the second from January 2021 to August 2023.

**Findings:**

48 leprosy cases were reported throughout the study, 83.3 % of which were diagnosed as multibacillary. The first period had an average detection rate of 0.38 cases per 10,000 inhabitants, increasing to 1.19 cases per 10,000 inhabitants in the second period. Notably, there was a substantial shift in the degree of physical disability (GD), with more Grade 0 and Grade 1 disabilities observed post-active search. Main Conclusions: The study underscores the efficacy of active search strategies in early diagnosis, highlighting a 300 % increase in the annual average of diagnosed cases. This large number of detected cases demonstrates the high sensitivity of the LSQ. This approach significantly aids in uncovering hidden cases of leprosy, enhancing disease management and control in low-endemic areas indicating that the Ministry of Health should intensify leprosy control activities in these regions.

## Introduction

*Mycobacterium leprae* and *Mycobacterium lepromatosis* causes leprosy, a chronic infectious disease that can cause permanent physical disabilities and several deformities, if not treated.^1^ Thus, the control strategy through early diagnosis is one of the pillars for controlling the disease.^2^ However, the long incubation period, generally with an average time of 2–7 years and which has been reported to reach 30–50 years in some studies, is a considerable limitation to achieving this goal.^3^^,^^4^ Leprosy is classified as a significant public health issue. In 2019, more than 200,000 new cases were reported worldwide, and 27,864 were reported in Brazil, accounting for 93 % of all cases in the Americas region and 13.7 % of the global cases registered. The impact of the COVID-19 pandemic in 2020 resulted in a 37 % reduction compared to 2019.^5^ According to World Health Organization (WHO) goals, it is necessary to scale up leprosy prevention alongside integrated active case detection, making our work valuable for meeting the goal proposed by the WHO.

Brazil is the country with the highest leprosy detection rate in the world. However, several problems are encountered in assessing and diagnosing cases in various regions of the country, especially in areas of low endemicity.^6^ Santa Catarina is a state in the South Region of Brazil has the fourth-highest number of patients with Grade 2 disabilities (G2D) in 2022, indicating a high risk of physical disability persists with nerve function impairment definitely (sensory, motor, or both) and visible deformities after treatment.^7^ It is believed that the higher the proportion of patients with G2D, the greater the delay in diagnosis and contribution to continuous transmission. Also, there is an increase in the proportion of patients with disabilities in low-endemic areas due to lower awareness and skills among health professionals.^8^ Moreover, Santa Catarina is the second with the lowest number of cases of leprosy in Brazil. In 2021, Santa Catarina notificated 136 new cases, corresponding to a detection rate of 1.85 cases/100,000 habitants.^9^

This study evaluates implementation strategies for leprosy diagnosis based on active search for leprosy diagnosis and screening practices of household contacts for scale up leprosy prevention alongside integrated active case detection. Thus, the study was carried out mainly in the municipality of Caçador which is located in the Alto Vale do Rio do Peixe region, in the Midwest of Santa Catarina, Brazil. One characteristic is the high turnover of health professionals with little continuing education training in the last ten years.^10^

Our study aimed to describe the epidemiological profile and evaluate the active search strategy and compared with passive case detection in a municipality in South Brazil, where the disease is not highly endemic. We analyzed data from two separate leprosy surveillance periods to gain insight into the operational situation of the disease in the area.

## Materials and methods

### Ethics and consent

This study was approved by the Research Ethics Committee of the Central Education Unit FAEM Faculty (protocol number CAAE 58925522.8.0000.8146, May 31st, 2022). It evaluated the active search strategy implemented in Caçador in 2021, so there was no need to sign an informed consent form to evaluate the medical records of diagnosed patients.

### Characterization of place

Caçador is a city in the interior of Santa Catarina, located in the stateʼs Midwest. It has a humid subtropical climate and an altitude of 920 m. The city covers an area of 981,910 km^2^ and has a population of 73,720. The cityʼs economy revolves around extractivism. It is considered the “city of wood” of Santa Catarina. The total GDP is R$ 2654,378 reais and the GDP per capita is R$ 58,773.87 reais. The constant economic growth is reflected in an HDI of 0.735, but with a Gini index of 0.50.^11^ Caçadorʼs healthcare situation, while showing some positive aspects, also highlights areas for improvement. The cityʼs average infant mortality rate is 15.07 per 1000 live births. In 2009, the city had 19 health facilities, indicating a need for further healthcare infrastructure. Only 51.21 % of the population is served by Community Health Agents, and Primary Care coverage is 52 %, suggesting a need for increased access to healthcare services.^12^

### Application of the leprosy suspicion questionnaire (LSQ)

**Fig. 1:**
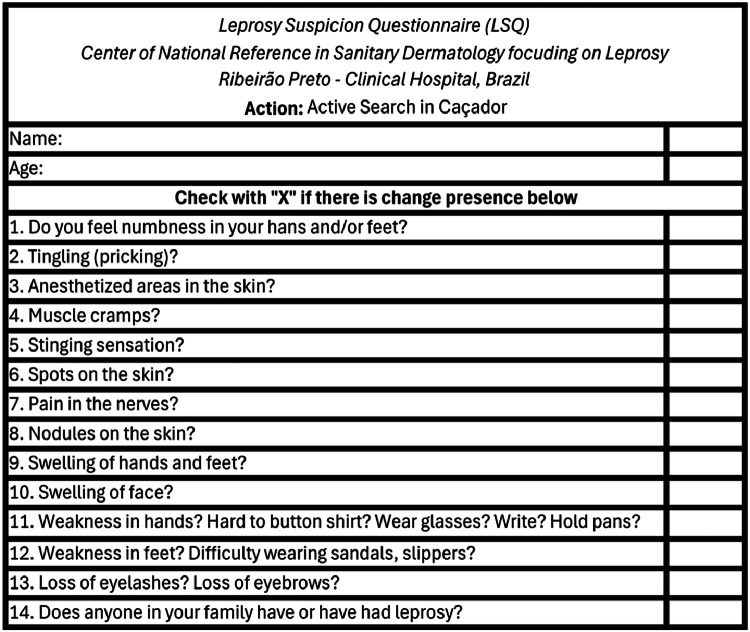
Leprosy Suspicion Questionnaire (LSQ).

The “Leprosy Suspicion Questionnaire” (LSQ), a screening tool created by the team at the Ribeirão Preto Medical School of the University of São Paulo and validated by Bernardes Filho et al. (2020). LSQ posed 14 simple questions about possible symptoms and signs related to leprosy (Fig. 1). In 2023, this tool was recognized as one of the principal active search strategies by the Brazilian Health Ministry.^13^

### Methodological design

Active or passive case detection can identify new leprosy cases. The period from 2014 to 2020 was characterized by passive case detection, meaning the patients had to go to health service locations. From August 2021 to August 2023, Caçador municipality in the Midwest of Santa Catarina, Brazil, trained primary care professionals to implement an active case search strategy through the infectious disease outpatient clinic.

In January 2021, a training course on managing and diagnosing leprosy in primary care was offered to doctors and nurses in the municipality's primary care units. The LSQ was presented at this training. It was the first stage of the project carried out by the municipality. This first stage was an informative course, showing primary care professionals the tools that would be used to find new patients.

In June 2022, the second stage of the active search for leprosy project was carried out in the municipality, with training for Community Health Workers (CHWs). A lecture was given on the signs and symptoms of leprosy, and the LSQ was implemented as a tool to be used during the CHWs' home visits. In July 2022, the CHWs began administering the LSQ in their community. Over a period of six months, a total of 207 forms were submitted to the infectious disease outpatient clinic. Of these, 152 forms (73.4 %) were found to have at least one positive response.

It is an essential report that the infectious disease outpatient performed the active search project; the infectious disease doctor and nurse did all the evaluations. No patient of the active search project was diagnosed by primary care. Only the infectious disease outpatient was working on the project. For this reason, the evaluation of the patients followed the rules. To identify potential patients, the clinic staff first examined 15 patient files with a known “family history of leprosy”. Following this initial evaluation, patients experiencing symptoms such as “nerve pain”, “cramps”, “weakness in the hands or feet”, and “skin blemishes” were contacted, and a total of 47 patients were selected.

The nurse and doctor in charge at the clinic then assessed these 62 patients in person. After the consultation, the patients were categorized as suspected, confirmed, or discarded cases based on their symptoms and medical history.

## Results

### Epidemiological profile of the patients

During the study period, Caçador reported 48 cases of leprosy. The average case detection rate for the period was 0.62 cases/10,000 inhabitants, with the average for the 2014‒2020 period being 0.38 cases/10,000 inhabitants, while that for the post-active search period was 1.19 cases/10,000 inhabitants. In addition, the average number of annual cases from 2014 to 2020 was 3 cases per year, while in the other period, it was 9 cases per year. Table 1 shows the other socio-epidemiological characteristics between the periods and whether there was a significant difference between them, determined using the *χ^2^* test. We can note that after the active search, there was a significant change in the standards of clinical classification and grade disability. There was a reduction in the number of cases with Grade 2 disability and lepromatous patients.

**Table 1:** Epidemiological profile of cases detected in Caçador, Santa Catarina, Brazil.

Category	Subcategory	2014–2020	2021–2023	*p*-value
		n (%)	n (%)	
Sex	Male	7 (33.3)	8 (29.6)	0.784
	Female	14 (66.7)	19 (70.4)	
Age	< 15y	0	2 (7.4)	0.085
	18 to 30y	0	5 (18.5)	
	31 to 40y	4 (19)	3 (11.1)	
	41 to 50y	9 (42.9)	4 (14.8)	
	51 to 60y	4 (19)	6 (22.2)	
	> 61y	4 (19)	7 (25.9)	
Years of schooling	Illiterate	2 (9.5)	0	0.060
	≤ 8y	14 (66.7)	12 (44.4)	
	9 to 12y	5 (23.8)	12 (44.4)	
	> 12y	0	3 (11.1)	
Classification	Indeterminate	2 (9.5)	1 (3.7)	**0.021**^a^
	Tuberculoid	4 (19)	1 (3.7)	
	Borderline	7 (33.3)	20 (74.1)	
	Lepromatous	7 (33.3)	2 (7.4)	
	Primary Neural	1 (4.8)	3 (11.1)	
Grade Disability	0	7 (33.3)	7 (25.9)	**0.025**^a^
	1	4 (19)	15 (55.6)	
	2	10 (47.6)	5 (18.5)	

a Statistically significant determined by using the Chi-Squared test. Y, Years.

### Analysis of the active search

As stated in the methodology section, 15 individuals who said they had a relative with leprosy were selected to start the investigation. The team evaluated only six individuals, of which three were diagnosed with leprosy. Of the 47 patients who called for an investigation appointment, 30 were evaluated by March 2023. Of these patients evaluated according to the four cardinal complaints of “nerve pain”, “cramps”, “weakness in the hands or feet”, and “spots on the skin”, 14 were diagnosed with leprosy. In total, 62 questionnaires were selected to start the investigation, but only 36 (69.3 %) patients were evaluated by the nurse and doctor. Among the 36 respondents, everyone answered/filled 5.6 questions on average and their distribution by the number of marked answers in both groups is described in Table 2.

**Table 2:** Number of individuals ranked according to total signs and symptoms of leprosy marked on the LSQ in order of frequency (*n* = 36).

Symptoms and Signs (LSQ+)	NLG, n	%	LG, n	%
1. Do you feel numbness in your hands and/or feet?	4	21.1	9	52.9
2. Tingling (pricking)?	3	15.8	8	47.1
3. Anesthetized areas in the skin?	0	0.0	4	23.5
4. Muscle cramps?	17	89.5	15	88.2
5. Stinging sensation?	5	26.3	6	35.3
6. Spots on the skin?	17	89.5	15	88.2
7. Pain in the nerves?	17	89.5	17	100
8. Nodules on the skin?	4	21.1	3	17.6
9. Swelling of hands and feet?	4	21.1	4	23.5
10. Swelling of face?	1	5.3	1	5.9
11. Weakness in hands? Hard to button shirt? Wear glasses? Write? Hold pans?	16	84.2	16	94.1
12. Weakness in feet? Difficulty wearing sandals, slippers?	4	21.1	4	23.5
13. Loss of eyelashes? Loss of eyebrows?	1	5.3	1	5.9
14. Does anyone in your family have or have had leprosy?	3	15.8	3	17.6
**Total number of people**	19		17	

LSQ, Leprosy Suspicion Questionnaire; NLG, Non-Leprosy Group; LG, Leprosy Group.

Considering the strategy of applying the leprosy suspicion questionnaires, 17 patients were diagnosed among the 152 LSQ+ individuals, with a new case detection rate of 11.2 %. Between 2021 and 2023, 27 patients were diagnosed with leprosy, 17 of them through active search. We compared the profiles of the ten patients diagnosed through passive detection with the 17 patients diagnosed through active search. The only statistically significant difference using the Chi-Squared test was Grade Disability, which showed that the active search patients had an earlier diagnosis than the passive detection patients.

## Discussion

The epidemiological profile of the cases notified before and after the active search was determined, and a significant difference was observed in the variables of clinical classification and the degree of physical disability. There was a 300 % increase in the annual average number of cases after the implementation of the strategy by the municipality. This indicates that there could be many patients who need to be diagnosed. Thus, during the years of study, 48 leprosy cases were reported, of which 83.3 % were diagnosed as multibacillary classification. 56.3 % of the patients presented the borderline clinical form and 18.8 % of lepromatous cases were diagnosed in a region considered to be low endemic. These data confirm the scenario of continued transmission, indicating a long delay for case detection, diagnosis and treatment, and its maintenance as a public health problem that has not yet been resolved.

The active search strategy carried out by the municipality of Caçador through the application of the LSQ by the CHWs managed to identify many cases that had gone unnoticed by the family health teams. Fifty percent of the patients assessed had a history of contact with a family member with a previous diagnosis, which is in line with the literature regarding the increased risk of the disease in contact persons.^14^ In addition, 46.6 % of the patients who completed the LSQ were confirmed to have leprosy according to the four cardinal signs chosen to start the active search, they are “nerve pain”, “cramps”, “weakness in the hands or feet”, and “skin blemishes”. This large number of cases demonstrates the high sensitivity of the LSQ, the greater the number of symptoms reported.^15^^,^^16^ In addition, the strategy applied by the CHWs was in line with the work of Bernardes Filho et al. (2021), who reported a diagnosis rate of 13.4 % using the same strategy of our study^17^ However, the author evaluated almost 30 % of patients with LSQ positive; in our study, we evaluated only 23.7 % of patients with LSQ positive. Other studies have evaluated the application of LSQ in closed prison systems, showing a lower detection rate in these places.^18^^,^^19^ In our work, the overall detection rate of leprosy patients by the total number of patients assessed in the period was higher than that of other active search work. We attribute this to the initial profile of the patients whom the health professionals would examine. That is, patients in contact with a leprosy patient and patients with four neurological cardinal signs. We expect the detection rate to decrease by expanding the search to patients with only one or two positive responses on the LQS.

The direction of public policies to combat leprosy is based on studies of highly-endemic locations, which allow the disease to be associated with social factors linked to poverty and vulnerability.^10^, ^11^, ^12^, ^13^, ^14^, ^15^, ^16^, ^17^, ^18^, ^19^, ^20^, ^21^, ^22^, ^23^ However, these associations are questionable, given the lack of knowledge about the actual prevalence of leprosy in these places. This study found no association between the epidemiological variables of the patients before and after the active search, which is in line with the results of studies in areas of low endemicity.^24^^,^^25^

For the years in which the active search for cases occurred (2021–2023), a higher number of cases with physical disability Grade I were reported. This can be explained by the greater focus on differential diagnoses of neural conditions and not just dermatological complaints. A study in Ethiopia also reported a higher number of cases with a degree of physical disability, even though the patients did not report any significant complaints at the time.^25^ The study by Bernardes Filho et al. (2021) also showed that almost 60 % of individuals diagnosed with leprosy during the active search had some degree of physical disability. Our study highlights the importance of neurological complaints for the differential diagnosis of peripheral neuropathies. The leprosy suspicion questionnaire was implemented as an active case-finding tool in Brazil in January 2023. Its focus is on neurological symptoms, and some studies have already shown its importance, especially in finding patients with neurological symptoms and Grade 1 disability.^17^, ^18^, ^19^^,^^26^

Passive surveillance of leprosy cases is ineffective in disease control, generating a more significant number of hidden cases.^27^ In 2022, only two out of ten patients diagnosed were referred for confirmation, while the other patients were directly called to the specialized service. Raising awareness among health professionals and patients is extremely difficult in the study of low-endemic countries, as many believe the disease no longer exists.^28^ Other variables should be added to increase early diagnosis, such as nerve ultrasound and serological screening with anti-PGL-I. Ribeiro et al. (2021) showed that spatial analysis associated with serological screening with anti-PGL-I is an essential tool in understanding the evaluation of health services and tracking index cases. Similarly, Silva et al. (2021) confirmed the importance of anti-PGL-I as a marker of the presence of the bacillus in the patient's environment. In addition, using RLEP molecular biology to assess asymptomatic or oligosymptomatic contacts has already been shown to be an important tool in deciding whether to provide early treatment.^18^

We understand that implementing the strategy based on theoretical and practical training, administering the LSQ, and providing diagnostic support increased the number of annual cases by 300 % in a low-endemic area. We consider this increase to be evidence not of the actual number of cases in Caçador but rather the result of a better detection rate due to the active search strategy. Although this statement covers the active search for a large part of the population, we are aligned with the Ministry of Healthʼs strategy of expanding diagnoses through Primary Health Care. We believe the LSQ should be used as a pre-consultation screening tool in primary care. New screening and diagnostic tools should be implemented in active searches, such as screening school populations to identify new cases in the home environment. In addition, disseminating knowledge to the population and health professionals and empowering community health workers in the search for suspected cases are essential for changing the reality of late diagnosis in low-endemic areas.

## Ethical approval and patient consent

This study was approved by the Research Ethics Committee of the Central Education Unit FAEM Faculty (protocol number CAAE 58925522.8.0000.8146, May 31st, 2022). This study evaluated the active search strategy implemented in Caçador in 2021, so there was no need to sign an informed consent form to evaluate the medical records of diagnosed patients.

## Funding

This study was supported by FAPESC (Notice 15/2021 ‒ Term of Grant 2021TR001301).

## Conflicts of interest

The authors declare no conflicts of interest.
